# Improving Vegetable Safety in China: Does Co-Regulation Work?

**DOI:** 10.3390/ijerph18063006

**Published:** 2021-03-15

**Authors:** Lita Alita, Liesbeth Dries, Peter Oosterveer

**Affiliations:** Social Science Group, Wageningen University & Research, 6700EW Wageningen, The Netherlands; liesbeth.dries@wur.nl (L.D.); peter.oosterveer@wur.nl (P.O.)

**Keywords:** food safety, co-regulation, pesticides residue

## Abstract

In the last decade, vegetable safety issues have received growing attention from both consumers and public authorities in China, as vegetable safety hazards pose a serious threat to public health. In 2017, the Industry & Trade Bureau in China implemented a “Market Renovation Program”. This program includes the renovation of wholesale and wet markets, the formal registration of all stallholders in these markets and the introduction of a rapid test for pesticides residues. We apply the co-regulation framework to assess the implementation and results of the renovation program on the safety of vegetables. A mixed methods approach is used to investigate the effects of the renovation program. The qualitative study elaborates on the implementation of the renovation program and the behavioural changes of stakeholders in handling vegetables through interviews and field observations. The quantitative results confirm that the renovation program has a positive impact on vegetable safety. In conclusion, this study shows that the key factor for the success of the renovation program is the transition of authority from the local, public authority to the market management.

## 1. Introduction

In the last decades, consumer demand for food safety has increased dramatically in China due to urbanization and a rising middle class. Vegetables are among the most important food items for Chinese consumers. Vegetable safety has therefore received growing attention, as vegetable safety hazards pose a serious threat to public health. Recent studies show that pesticides residues are the most prevalent safety hazard in fresh vegetables [1,2]. Many different highly toxic pesticides including chlorpyrifos, carbofuran and omethoate are prevalent in China [3]. Exposure to pesticide residues may lead to multiple hazards ranging from short-term/acute problems (e.g., skin and eye irritation, headaches, dizziness, and nausea) to chronic impacts (e.g., cancer, asthma, and diabetes) [4]. Previous studies on consumer perceptions highlighted concerns among consumers over food safety [5]. For instance, research has found that the main reason for Chinese consumers to buy organic food is to avoid pesticide residues and other food safety hazards, while consumers in other countries mainly buy organic food to minimize the environmental impact in agricultural production [6,7]. Similar results were found in studies on consumer preferences over different retail formats, namely, that the choice of retailer by Chinese consumers is mainly driven by food safety concerns [8,9].

Public authorities have also attempted to improve food safety by labelling agri-food products and by restructuring the food supply chain. In 2003, the Ministry of Agriculture introduced the “Food certification label policy (*Nong Chan Pin Zhi Liang An Quan Ren Zheng*)” in China, which classifies vegetables into three standards: hazard-free, green and organic. In Europe and North America, food certification and classification have proven to be an effective way of controlling food safety hazards [10,11]. However, monitoring small-scale vegetable farmers in rural China is difficult. Recent studies have found that both consumers and producers lack trust in and recognition of the certification policy [12,13].

In addition, lead firms in the retail sector have started to integrate the food supply chain in an attempt to improve food safety. More specifically, the expanding large-scale supermarket sector in China is expected to improve food safety by enforcing corporate (private) food safety standards [12,14]. To avoid food poisoning incidents and scandals, supermarkets have established their own production bases where farming and harvesting practices are guided by detailed corporate standards [14]. However, researchers have also expressed concern about the intensive farming and long-distance transport that are characteristic of this supply chain integration [15,16]. Moreover, previous research may have overestimated the impact of supermarket expansion on the vegetable supply chain in China. Recent studies have found that large-scale supermarkets, including Walmart, still purchase a substantial share of their vegetables from external suppliers, i.e., small-scale wholesalers [14,16]. The ability of the large-scale supermarket sector to prevent food safety problems may therefore be limited.

The amendment of the “Food Safety Law” in 2015, is the most recent regulation concerning food safety control in China [1]. The new food safety law authorizes the Chinese Food and Drug Administration (CFDA) and the Industry and Trade Bureau (ITB) to oversee food safety in China and confirms the legal liability of actors in the food supply chain for food safety hazards. However, the CFDA and ITB can only regulate food retailers, who are primarily located in urban areas. Because of the dual rural-urban administration system in China, the CFDA and ITB have little control over the practices of farmers in rural areas who also affect food safety, such as the use of pesticides, nor over the actions of wholesalers who transport fresh food from rural to urban areas [17,18]. Rural governments have used extension services to guide farmers in the proper application of pesticides but with only limited success. The main cause for this limited success has been the limited financial and human resources available to local governments to reach the Chinese small farmer population of more than 200 million households [8].

In comparison to China, the European Union and the United Kingdom have developed regulatory frameworks that enable public-private collaboration to oversee food safety controls. In 2002, the EU introduced the General Food Law and established the European Food Safety Authority (EFSA) [19,20]. Under the General Food Law, the EFSA was established as an independent authority to undertake risk assessment in the EU. Within this framework, the private sector is primarily responsible for ensuring the safety of food products, while public authorities in the member states provide oversight over the private actors at all stages of production and distribution [21]. The food safety law of the UK took private sector responsibility even further and led to the development of private farm assurance schemes to implement the official code of practice and guarantee food safety along the food chain [22].

Since 2006, the EU Commission and member states have developed strategies to reduce the impact of pesticides application on consumer health and the environment. Regulation of the market for plant protection products and the directive on the sustainable use of pesticides provide the regulatory framework and practical guidance for farmers to improve the quality and efficacy of pesticide application equipment, to ensure better training and education of users and to develop integrated pest management schemes [23].

To mitigate the potential for food safety crises in China, public authorities have started to intervene at the market side of the vegetable supply chain in 2017. The main regulator for urban food provision is the State Administration for Industry and Trade (ITB). The bureau has subdivisions at the provincial, city and county level. In 2017, the Industry and Trade Bureau of China introduced the “Wholesale and Wet Market Renovation and Upgrade Policy (*Gui Fan Hua Nong Mao Shi Chang*)” (known as the renovation policy). This policy aimed to improve food safety in wholesale and wet markets by renovating their infrastructure.

The renovation program emphasizes the need for “collaboration between public and private” and “co-regulation with stakeholders”. Such a framework of public-private co-regulation proposes that the responsibility of food safety control should be shared among the actors in the supply chain and that safety hazards can be prevented by measures such as market incentives, education and training [24]. The co-regulation framework was introduced in the early 2000s for food safety management in Europe and North America [21,25]. To include the private stakeholders in the regulation, the co-regulation framework emphasizes transparency, capacity building and market incentives. Co-regulation is feasible if the public authority can select policy instruments suitable for different stakeholders. The EU-coordinated control programme is viewed as an excellent example of a co-regulation approach. The European Food Safety Authority has created a farm-to-fork regulatory framework and a science-based risk assessment process by collaborating with member states as well as with private stakeholders in the supply chain [19]. In recent years, Chinese researchers also point to co-regulation as a potential solution for food safety governance because fragmented and underdeveloped regulators cannot adequately monitor all smallholders and hence cannot prevent food safety issues at the source [18,20]. Thus, they suggest sharing the responsibility for food safety and provide incentives for the private sector to guarantee food safety.

This study intends to shed more light on how the renovation policy has affected different stakeholders in the market, an issue that has been absent in most research. Moreover, to date, it remains unknown whether the renovation program indeed improved vegetable safety in wholesale and wet markets. Therefore, we specify our research question as follows: did the renovation program successfully enhance food safety in agri-food markets; and if so, how did the renovation program achieve the improvement and what role did the stakeholders play in this process. Using both qualitative and quantitative research methods, this research investigates changes at the macro-institutional level as well as at the micro-behavioural level. We use food safety inspection data from the Chinese Food and Drug Administration (CFDA) before and after the implementation of the market renovation program to determine the impact. The rest of the paper starts with further introducing the conceptual framework of co-regulation, followed by a discussion of the research methods. Section 4 presents the findings from our research, followed by a discussion of these findings in Section 5 and a conclusion.

## 2. The Co-Regulation Framework

A food safety crisis is acknowledged as a threat to public health. Therefore, public authorities are considered responsible for overseeing the safety of food. Nevertheless, food is produced and supplied by the private sector through markets. Public authorities endeavour to reduce food safety hazards by reinforcing controls and regulations [20,24,26]. However, research has found that regulation cannot be effective without the participation and commitment of the private sector [27,28]. Mitigating food safety issues, therefore, requires the commitment of both public authorities and the private sector. In recent decades, various co-governance frameworks have been developed for the provision of common goods [29]. In the 1990s, the theory of social governance focused on the construction of a collaborative network to analyse the collaboration between diverse actors [29]. Later, researchers introduced the concept of “co-regulation” in food safety control. Co-regulation is defined as “a dynamic interaction between public and private actors to secure the provision of common goods” [19,29].

Food safety risk co-regulation is the process by which participating actors cooperate using government regulation, market incentives, technical regulation, social supervision, and information dissemination [20,28]. Fearne and Martinez introduce the co-regulation framework to combine public intervention and self-regulation. They define food safety co-regulation as *“the process by which the government and companies cooperate to construct an effective food safety system to ensure better food safety…, under the premise that all stakeholders in the food supply chain (from production to consumption) can benefit from improved governance efficiency”* ([21], p. 2). They reviewed the development of food safety control in North America and Europe and concluded that by including the private sector in the standard setting and by sharing responsibility and authority with stakeholders, a higher level of efficiency in food safety governance was achieved.

Generally, co-regulation consists of four stages in the regulatory process: (i) setting food safety standards; (ii) implementation; (iii) enforcement; and (iv) monitoring [19]. First, the co-regulation framework promotes transparent information sharing and comprehensive consultation instead of top-down standard setting when creating new agreements, conventions and laws that relate to food safety. More specifically, Fearne and Martinez argue that private stakeholders should have the right to be informed about different policy options and the policy decision-making process [21]. Second, the co-regulation framework encourages private stakeholders to implement their own internal rules, as long as they uphold the agreed-upon food safety standards. Instead of direct regulatory control, a self-regulatory approach is proposed to manage food safety within firms. Third, before the introduction of co-regulation, food safety standards were primarily enforced by seizing products and closing facilities. However, the experiences in Europe and North America show that enforcement can take the form of promoting good practices through advice and education rather than enforcement action. In addition, market-based reputational mechanisms can enhance the compliance with food safety standards. Finally, regarding the monitoring of compliance, the co-regulation framework demands a shift from direct inspection to performance evaluation and suggestion. In short, the renovation program granted new requirements and responsibilities to both public authority and private stakeholders.

## 3. Research Methods and Location

This study applies a mixed method approach with a focus on in-depth qualitative research. The fieldwork was implemented in Shandong Province. Shandong province is one of the main vegetables producing and consuming provinces in China. The economic development of Shandong (GDP per capita in 2017: 10,303 U.S Dollar) is slightly above average in China (8643 U.S Dollar) and ranks tenth out of the 31 provinces. Shandong province has a population of 98 million people, of which 57% live in urban areas [30]. There are 17 cities in Shandong province.

To estimate the results of the renovation program, four cities in Shandong province (Jinan, Qingdao, Weihai and Zibo) were selected. Data was gathered from the food safety risk monitoring system on the official website of the Chinese Food and Drug Administration (CFDA) (further information can be found at http://samr.cfda.gov.cn/WS01/CL1667/index_5.html accessed on 31 December 2017). The data are the result of a systematic collection of monitoring data and related information on foodborne diseases, food contamination and food hazards from food samples at the market and retail level. The officially published reports contain detailed information on the type of food safety hazard, maximum residue levels (MRL) and inspection results. Like the European Food Safety Authority (EFSA), the CFDA applies a general default MRL of 0.01 mg/kg [31]. The main purpose of risk monitoring is to detect food safety hazards through randomly drawn representative samples from food sellers. The data are representative of the general food safety situation in China. Because of the limits of using secondary data, we use the CFDA reports merely to complement the qualitative study. It is beyond the scope of this paper to conduct an empirical investigation that directly tests the causality between the renovation program and food safety.

CFDA reports contain the following information: food safety status (contamination detected or unsafe/no contamination detected or safe), name and address of the seller as recorded during the inspection, and date of purchase. We establish a dataset by retrieving all observations on vegetable tests from wholesale and wet markets in the four cities in Shandong province. Our dataset contains 2784 samples of tested vegetables, and each observation is the outcome of one test on a specific vegetable in a specific market. We define food safety as a binary variable: the sample is reported as unsafe if the pesticide residue exceeds the MRL and is considered safe otherwise. The dataset was retrieved from the CFDA reports in 2016, 2017 and 2018. The renovation program was implemented in October 2017. The reports from 2016 and from 2017 between Jan. to Sept are categorized as “before renovation”. The reports in 2018 are considered as “after renovation”. We used the list of renovated markets released by the ITB to identify which markets in the dataset were renovated and which were not. We found that 1191 observations came from these renovated markets. Further information can be found in the Appendix A. Because renovated/nonrenovated and safe/unsafe are binary variables, we add Chi-Square tests to check the significance of differences in safety levels between selected/non-selected markets and before- and after-renovation.

We select two cities: Jinan and Qingdao, for qualitative investigation using in-depth interviews and field observation. We expect that the implementation of the renovation program, and hence its effects, differ between cities. Considering that the renovation policy was issued by the central government but implemented by the city-level ITB, it is expected that the provincial capital city will receive more attention. In addition, the ITB in the capital city might possess more financial and human resources to implement the renovation program. The peripheral cities may lack resources and pressure to renovate markets. Thus, the renovation may have a smaller impact on the peripheral cities. Before we can claim that the renovation program has succeeded, we need in-depth interviews and field observation to verify the results and to clarify the mechanism behind this renovation.

In Jinan, 16 markets were selected for renovation. These 16 renovated markets consisted of eight wholesale markets and eight wet markets. Qingdao is a coastal city, where 14 markets were renovated (eight wholesale markets and six wet markets). In these two cities, 14 markets were visited during the research. The differences between renovated and non-renovated markets were observed. The differences in market infrastructure before and after renovation were recorded by interviewing staff. Wholesalers and retailers were interviewed to assess their behavioural change due to the renovation. In total, we randomly selected 56 wholesalers and stallholders and conducted interviews with them. As shown in Table 1, different types of wholesale and wet markets are covered in the fieldwork. The selection of the interviewees was based on two characteristics: the market level and the participant level. Markets are distinguished as wet markets and wholesale markets; and as renovated markets and unrenovated markets. We have visited each type of market to ensure the representativeness of this study. Hence, we visited three renovated wet markets; six renovated wholesale markets; two unrenovated wet markets and three unrenovated wholesale markets. At the participant level, we interviewed wholesalers and retailers in each market. We asked open-ended questions until no new information was found. In the fieldwork, we found that conducting four interviews per market was appropriate for collecting information about the participants’ daily routines. Nevertheless, accessing and interviewing the market management is a more challenging task. We successfully interviewed 5 market staff in the renovated markets, of whom 4 are from Jinan and 1 from Qingdao. However, the management of unrenovated markets refused all external visitors. In general, we have a comprehensive overview on the participants of the renovation program.

To secure the external validity of this study, we visited markets in different cities. The type, size and location of the markets vary, as well as the size and background of the wholesalers and retailers that were interviewed. For internal validity, a clear narrative from the policy document to safety inspection was created. The steps were elucidated following this structure: the announced renovation plan, the change in infrastructure following the plan, the behavioural change due to the new infrastructure, and the change in food safety levels brought by the change in behaviour.

## 4. The Implementation of the Renovation Program: Towards Co-Regulation

### 4.1. The Renovation Program

The formal document of the renovation program considers the market management and tenants of wholesale and wet markets as key stakeholders. The renovation program for wholesale and wet markets that was released by the State Administration for Industry and Trade in 2016 includes five measures:

- The market management should provide training for tenants. Tenants are required to learn the Food Quality and Safety Protocol before they enter the market. The protocol indicates the food safety standards and penalties for food safety hazards.
- Tenants must register with the market management if they want a place in the renovated market. The market management requires tenants to provide a copy of their ID card for traceability.
- Tenants are required to provide certification of the origin of the vegetables (including details of the producer and harvest time) for traceability.
- The market management should set up a rapid testing room with specialized staff. Vegetables in the market should be regularly tested.
- The market should keep records of the test results and set a display screen to announce the test results for consumers.

The renovation program was implemented under the instruction of the city administration of industry and trade. The city ITB selected the markets to be renovated by the end of 2017.

### 4.2. Actors in the Renovation Program

**Figure 1:**
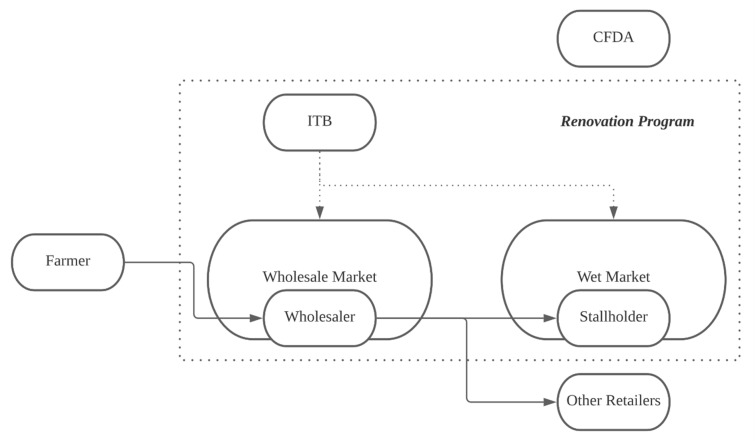
Actors in the vegetable provision system and involved in the renovation program. Note: CFDA stands for the Chinese Food and Drug Administration. ITB stands for the Industry and Trade Bureau.

Generally, the renovation program of markets in China involves three actors: the local policy maker as overseer of the market, the wholesale and wet market management, and the wholesalers and stallholders as tenants of the market. In practice, the renovation program can be seen as an intervention guided by the local ITB, implemented by the wholesale and wet market management and modifying the behaviour of the wholesaler and stallholder. This section discusses the role played by these actors. Figure 1 illustrates the actors in the vegetable provision system and whether they are included in the renovation.

Conventional markets in urban China can be distinguished into wet markets and wholesale markets. A wet market is a location for the public gathering of buyers and sellers at a known time. Wet markets contain a limited number of stallholders (10–30) and provide fresh food for consumers living nearby. Wet markets involve a large number of transactions of relatively small quantities of goods on a face-to-face basis between a seller and buyer. Sellers in a wet market are commonly designated as stallholders because they rent a market stall to display, advertise and store their goods. Stallholders in wet markets normally operate independently on an individual or family basis. The types and quantity of vegetables are decided by the stallholders themselves. In the 1990s, the stallholder and market owner (enterprise or collective) maintained a simple tenant-landlord relationship [32]. In the last decade, urban municipalities and consumers have raised demands on the environment of wet markets. As a result, the market owner is required to hire security and cleaners to preserve order in the market [33]. Moreover, the market owner is responsible for reconciling conflicts between consumers and stallholders. For this purpose, the market owner must hire specific personnel and market management. Through this process, the market and stallholders have established a more coordinated relationship.

Wholesale markets are usually located in urban areas. The wholesale market provides space for hundreds of wholesalers to display and store their goods. Normally, stallholders from the wet market and other retailers visit the wholesale market in the morning and buy in bulk. Without wholesalers, retailers would need to purchase directly from farmers, which would involve many minor transactions [34]. Through the wholesale market, the number of transactions is reduced, and the marketing process is simplified. In this case, the retailer does not need to be concerned with any of the sorting, reassembly or distribution functions and concentrates solely on selling to consumers. Wholesalers shuttle between farmland and urban areas, purchase vegetables in small quantities from farmers, aggregate vegetables in bulk and bring them to the wholesale market. Because most wholesalers drive their vehicles into the market, they need to check in at the entry gate. An entry toll is usually paid at this point based on the size of the vehicle and an estimated volume or a weight established from passing over a weighbridge. After the check-in, wholesalers unload the goods at a space allocated to them by the market management. Large-scale wholesalers that can afford the rent will store and display their goods in a permanent shopfront. Other wholesalers park their vehicles in the covered space. In the 1990s, wholesale markets were merely open spaces for parking vehicles and exchanging goods [34]. In the last decade, wholesale markets have experienced similar adjustments as wet markets. Market management and regulation have been established in both wholesale and wet markets, and wholesalers must comply with the requirements of the market management.

As tenants, wholesalers and stallholders are both regulated by tenancy agreements with the market management. These tenancy agreements are publicly accessible in the market. Furthermore, tenancy agreements have similar terms in different markets, emphasizing fire drills, fair trade and fraud prevention. Sanctions such as penalties for violating these clauses are announced publicly. To enforce market regulation, market management has the power to punish and reward tenants in the market. In the interviews, stallholders, wholesalers and market personnel mentioned that sanctions such as a penalty or cancellation of the tenancy agreement are rarely implemented in practice. Nevertheless, the market management holds many tools to reward tenants and can thereby influence their behaviour. For instance, the stall closest to the market entry is preferred by tenants because of the higher turnover. Some tenants are offered better and larger positions in the market because they comply with the market rules. In addition, tenants are offered rent discounts in the following year if they can continuously comply with the market rules in the current year.

Market management and tenants are expected to provide agri-food products free of food safety hazards. In every wholesale and wet market, the market management demands tenants to guarantee the safety of their agri-food products. The importance of food hygiene and absence of food safety hazards are emphasized in the tenant agreement. Agri-food products are exposed to chemical contamination during production, transportation and storage. Vegetables can be contaminated at any stage before they reach the consumer. Thus, vegetable safety requires engagement of the wholesaler, stallholder and market management. Nevertheless, without institutional guarantees, tenants and market management lack the tools and incentives to ensure food safety.

The ITB Department of Market Regulation is responsible for supervising wholesale and wet markets and other food trading entities. This department controls general market transactions in China, through drafting and implementing measures regarding the market order, regulating online transactions in goods and services, administrating supervision over contract execution, investigating and punishing illegal acts such as contractual fraud, taking charge of chattel mortgage registration, regulating brokers and auctions, organizing and instructing the credit rating of commodity transaction markets, and initiating special campaigns to address ongoing marketplace problems.

Within this extensive domain of the ITB, food safety issues were not their primary concern in the past. The relationship between markets and the bureau has changed dramatically in recent decades. In the 1980s and 1990s, most markets were established and managed by the local ITB and local municipalities. Later, markets were privatized and transferred to private corporations [35]. Because these corporations usually run multiple businesses in the city or province, complying with the ITB’s requirements is crucial for them. The scope of the ITB with respect to food safety ranges widely and includes general matters relating to cleaning, the disposal of waste materials, and detailed technical requirements for food hygiene (further information can be found at http://www.samr.gov.cn/spscs/ accessed on 31 December 2017). However, neither the ITB nor the market management had an inspection plan and protocol to survey and control food safety and quality before the market renovation policy in 2017. Until then, wholesalers and retailers had to apply for a food trade license to be permitted to trade food. However, due to the large number and the flexibility of the retailer and wholesaler, the bureau had difficulties controlling the quality and safety of their goods.

In 2016, the state administration released the executive order for a wholesale and wet market renovation program. Early 2017, the city-level ITB started approaching market management and selected the markets to be renovated. Later, in October 2017, the top-down renovation program was implemented. The wholesale and wet markets were required to maintain a high standard of food hygiene, employ their own staff of inspectors and establish a fully equipped and staffed laboratory. In the next section, we discuss the implementation of the renovation program in more detail.

### 4.3. Implementation of the Renovation Program

In general, the market management actively participated in the implementation and enforcement of the renovation program, leaving the standard setting and monitoring to the ITB. The infrastructure of the wholesale market and wet market has significantly improved after the renovation following the standards set by the ITB. As part of the implementation, large-scale wholesale markets, such as Qilipu, established special rooms and hired staff for rapid testing of food safety hazards. The room is at the entry of the market, and the staff takes samples from every vehicle that enters the market gate. The market management of Qilipu hired at least five people for the inspections while the smaller wholesale market, Huazhong, has only one inspector. Although the ITB provided equipment and instruction for staff, the market management is required to implement the renovation program and cover the costs. Large-scale wholesale markets can afford the costs of renovation and payment for additional staff. Smaller wholesale markets may lack the resources to comply with the requirements of the renovation program.

During the interviews, wholesalers were asked about what would happen if pesticides residues were found in the sample. The wholesalers in different markets provided similar answers: *“if the vegetables are contaminated and the contamination is detected, the whole batch of goods cannot be sold in the market. In addition, staff will ask us to remove the goods out of the market”.* This answer implies that the enforcement of the renovation program is the obligation of the market management. The stallholders in the wet market describe the procedure to be similar as in the wholesale market. The market management also mentioned penalties for selling contaminated food. However, none of the display screens in the markets that were visited actually worked. The management mentioned: *“the test results are recorded yet not accessible for external visitors”.*

The vegetables in the renovated markets are still not traceable. The wholesalers and stallholders can only provide general answers such as *“the cabbage was produced by a local farmer; the onion was delivered from Hebei province”*, etc. In addition, the sellers have received no training or instructions about vegetable safety or pesticides residues. The market management and staff for safety tests hardly communicate with the sellers. They believe that sellers sometimes spray illegal chemicals on vegetables to keep them fresh. One CFDA document also indicates that sellers were found to be using fungicides on vegetables during transport and storage.

The standard for the food safety hazards rapid test was issued by the State ITB without consulting the market management. The rapid test room aims to directly detect food safety hazards. The main threat for vegetable safety is pesticides residues. Thus, the rapid test aims primarily at detecting pesticides. Moreover, heavy metal pollution and other chemical contamination can also trigger the rapid test alarm. Pesticides include fungicides, herbicides, and insecticides. To detect pesticides residues, gas chromatography, high-performance liquid chromatography, capillary electrophoresis and mass spectrometry methods have been developed [36]. These methods are effective and precise, but they are also time-consuming and complex, and they require expensive instruments and highly skilled personnel [37]. Therefore, an enzyme-based biosensor (also known as the enzymatic method) was selected by the ITB as an acceptable alternative that is simple, rapid, sensitive, low-cost and reliable in the detection of pesticides (the enzymatic method was invented by Bean and Ankinson in 1964. In the 2010s, this method is mainly applied in China and India in on-site pre-tests for its convenience). The enzymatic method can detect almost all pesticides applied on vegetables, including parathion, malathion, methyl parathion and chlorpyrifos [38].

However, the enzymatic method has been criticized for its low accuracy. The official maximum tolerance level for pesticides is 0.01 mg/L. The enzymatic method can achieve a lower accuracy depending on the pre-test concentration and the skills of the staff. Hence, the enzymatic method can provide false negative results in practice, i.e., contaminated vegetables are likely to be omitted and considered as safe. However, the enzymatic method is unlikely to provide false positive results, i.e., mistaking safe vegetables as contaminated because only a high volume of pesticides will trigger the enzyme-based biosensor [37].

In conclusion, we find that three out of the five measures from the renovation program have been implemented. By following the standard issued by the ITB, market management set up a food safety hazards rapid test room and tests goods that enter the market. The test results are recorded for the local ITB. In every renovated market, the sellers are required to register with their ID card and pay the rent in advance.

Table 2 gives an indication that the renovation program has a positive impact on vegetable safety. Before the renovation, in 2016 and 2017, the share of unsafe vegetables in markets that were destined for renovation was between 12% and 14%. This was similar to detection rates in other markets. In 2018, after the renovation, the share of unsafe vegetables in renovated markets dropped to 7.45%, whilst the share in other markets increased to 16.67%. For the results in Table 2, the X^2^-coefficient is 341.331 (*p* < 0.001). This indicates that the renovation has significantly improved the safety level in renovated markets.

Table 3 exhibits the variation between cities. We assume that the success of the renovation program is highly dependent on the investment of the city-ITB. As we expected, the share of unsafe vegetables in renovated markets in Jinan decreases significantly after renovation compared to other markets, whilst this improvement in vegetable safety is not observed in Weihai and Qingdao after renovation.

In short, we found that the most significant measure of this renovation is the establishment of a rapid test room and the implementation of the comprehensive test. This rapid test was enforced and resulted in a lower detection rate of food safety hazards in wholesale markets. The success of the renovation is mainly the result of the collaboration between the ITB and market managements. First, the ITB offered clear instruction on the standard setting and training for the food safety test staff. Although we cannot confirm that the market management was consulted in standard setting, the city ITB selected the least complex and time-consuming test method, the enzymatic method, to ensure the renovation can be implemented and enforced. The main barrier for the ITB to enforce the food safety standards by inspecting every wholesaler directly is the mobility of wholesalers. Nevertheless, market management enforced the standard by using the dependency of the wholesaler on the market. In addition, the successful enforcement is the result of the authority transition from ITB to the market management. After the renovation, the market management was granted the authority to force the wholesaler to dispose of contaminated vegetables. Whereas the market management closely monitored the rapid test results, it remains unknown whether the monitor data was delivered to the ITB as well. At last, we found that the wholesaler was excluded from every process of the renovation program. The participation of the actors in the four co-regulation processes is presented in Table 4.

### 4.4. Implications of the Renovation Program for Wholesalers and Stallholders

Whereas the wholesalers were not consulted in the standard setting and received no food safety knowledge or training, their behaviour and routines were modified. Comprehensive inspection, meaning that every batch of vegetables is tested, results in significant changes for wholesalers. In wholesale markets, the tests can cover all goods because the wholesalers drive their vehicle into the marketplace through the market entrance. The market personnel describe the procedure before and after the renovation as follows: *“the wholesalers are required to stop at the entry. Their vehicles are weighted, and they need to pay the rent according to the weight of their goods. After the renovation, samples will be taken from their vehicle while the vehicle is being weighted. The driver is asked to park nearby the entry temporarily until the rapid test result comes out. If the result is positive, vegetables in this vehicle are not allowed to be sold in this market. The wholesaler must remove the goods”.* Buyers drive empty vehicles into the wholesale market to purchase vegetables. They can enter and leave the market without inspection because their vehicles are much smaller than the wholesalers’ and easy to distinguish.

Officially, the rejected vegetables are being disposed. However, the market management has no authority to oversee wholesalers’ activities outside the market. The interviews revealed several possibilities with respect to the rejected goods. The wholesaler has the right to apply for a reassessment. Their vegetables will be sent to a third-party inspection institute to acquire more accurate results. However, the staff of the market mentioned that they have never encountered this situation, possibly because the rapid test is likely to provide false negative results but unlikely to provide false positive results. Wholesalers are aware that the third-party test is very likely to reveal more hidden hazards such as the use of prohibited pesticides. When asked about the rejected goods, wholesalers claimed: *“we always dispose of the goods as formally required”.* The risk for wholesalers seems too high if they try to find buyers outside of the wholesale market. The market staff believed that *“the wholesaler attempts to sell the rejected goods to retailers to avoid loss”*. Nonetheless, the staff also acknowledged the difficulty of trading outside of the market. Typical stallholders in wet markets or retailers sell 20 to 30 types of vegetables in small quantities (10 to 20 kg) in their stall or store. However, a wholesaler sells one type or a few types of vegetables in large quantity (more than 1000 kg). Thus, a retailer needs to visit more than ten wholesalers to find all vegetable types and, likewise, a wholesaler must trade with dozens of retailers until all the goods are completely sold out. Thus, it is beneficial for retailers and wholesalers to assemble in a certain location to complete their transactions. Outside the formal wholesale market, it is impossible to find such a location in urban or suburban areas (it should be noted that this conclusion is based on interviews with small-scale wholesalers only because the large-scale wholesalers refused to answer questions about food safety hazards).

In response to the renovation, wholesalers have changed their behaviour to avoid being rejected at the market entrance. Wholesalers mentioned: *“we consider the issue of food safety more when buying vegetables from the producer”*. Before the renovation, wholesalers considered merely the price and freshness of the vegetables. As they know that contaminated vegetables will be rejected by the market, they have adjusted their buying behaviour to minimize the risk of rejection. In the interviews, wholesalers vaguely mentioned: *“we purchase from a ‘reliable source’ or a ‘local source’; we know which vegetables are safe for we have experience in producing vegetables”.* The personnel in the market agreed that *“many of the wholesalers used to be vegetable farmers themselves”.* Wholesalers may prefer to buy local vegetables because they are more familiar with local farmers and can check whether the farmer has a reputation of pesticides overuse, when the vegetables were harvested, and what the length of the post-harvest interval was. Pesticides residues on vegetables depend on the pesticide type, the amount that was applied and the post-harvest interval. If more information is available, the experienced wholesaler can select the vegetables that are most likely hazard-free. However, tracing the vegetables in the wholesale market back to the producer may not be possible due to the lack of information. In addition, another hidden activity was revealed by the market personnel: *“to avoid food loss, many wholesalers sprayed fungicides and other chemical stuff on the vegetables. After the rapid test was established, wholesalers stopped or at least reduced spraying fungicides on vegetables, for the risk of being detected is too high”.*

Not all wholesalers are equally dependent on the market and we observed clear differences between wholesalers. Large-scale wholesalers possessed sufficient human resources and equipment to maintain the safety of the vegetables that they sell even before the renovation. Small-scale wholesalers, on the other hand, may lack the resources and capacity to control food safety. The additional safety inspection established by the renovation program helped small wholesalers to detect contaminations and thereby enhanced their food safety level.

In addition to wholesale markets, the renovation program was also implemented in wet markets. Although both types are included in the renovation program, the impact of renovation might be different. The safety control in wet markets differs slightly from the control in the wholesale market because the stallholders have a different daily routine. Stallholders drive small vehicles to transport vegetables from the wholesale market to the wet market. After arriving at the wet market, they need to move their goods from the vehicle to their stall as quickly as possible. Unlike the large-scale wholesale market, most wet markets are located in urban areas with a high population density. Thus, asking stallholders to wait outside the market is impractical. The market personnel visit every stallholder’s stall and randomly take one or two types of vegetables to test. If the result is positive, the retailer is asked to dispose of this type of vegetable. The staff claims that: *“stallholders will normally comply because the value and quantity of one vegetable type in their stall is low*”.

The interviews with stallholders failed to find any remarkable change in behaviour: *“we buy and sell vegetables as we used to, with or without the rapid test”*. When the staff comes to collect samples for the rapid test, the stallholders simply comply. One staff member in the wet market said that *“some of the stallholders buy vegetables from the vehicles parked in the street for lower prices. The vegetables in these vehicles did not enter the wholesale market due to either low safety degree or to avoid paying the market fee. However, fewer stallholders choose to buy from the “street parking vehicles” nowadays thanks to the rapid test”*. The CFDA data supports our observation. Table 5 indicates a clear after-renovation difference between wholesale markets and wet markets. Whereas the selected wholesale markets reduced the detection rate from 13.66% to 3.43%, the renovated wet markets have a comparatively high detection rate 10.87%. The results for Table 5 show that the renovation did not significantly change the safety level in wet markets (*p* = 0.291). However, the renovation successfully changed the safety level in wholesale markets (*p* < 0.001).

## 5. Conclusions

The renovation program involves the public authority, market management and tenants. The program was implemented mainly by the ITB with the participation of market management. After the state administration issued the renovation plan in 2016, the city-level ITB was required to implement the policy. First, the city-level ITB selected some of the markets in the city and provided guidance on how to build the rapid safety check, the equipment for rapid testing, and instructions for the staff. The market management is required to build the safety check, display screen and entry control, and pay the staff’s salary. The wholesalers and stallholders were not consulted in the renovation policy. Nevertheless, the behaviour of the wholesalers has changed because of the recently established infrastructure.

This study reveals the important role of the wholesaler and the wholesale market in guaranteeing vegetable safety. We highlight that the vegetable safety level can be enhanced by developing a co-regulation strategy between private market management and local public authority. This study reveals that the key factors for the success of the renovation are the commitment of the local public authority, expansion of the market management domain, and the dependency of the wholesaler on the market. The conclusion of this study is relevant for the rest of China as well as for the Global South. In urbanizing areas, consumers raise higher demands for food safety. By reinforcing inspection and control in the marketplace, food safety hazards can be reduced. For the municipality, this study provides an additional solution for food safety issues besides retail modernization. Supermarketization is not the only possibility for developing the food retail sector. By transferring certain responsibilities to the market management, the local municipality can enhance the food safety level. The high dependency on the market for food retailers and wholesalers in cities in the Global South gives the management leverage to secure the quality and safety of the food they sell despite their flexibility and small scale. This strategy is particularly effective in wholesale markets.

Moreover, this study highlights the role of the “market management” as a relevant actor in the public-private co-regulation framework. In previous research, conventional wholesale and wet markets have been treated as an assembly of stallholders. However, the local municipality has empowered market management and supported its expansion in the regulatory domain. This has made the market management a comprehensive regulator that controls crucial resources for the provision of vegetables. The provider of such a location for trading holds an essential resource. For markets in urban China, this resource is the authorization given by the municipality. The permission of transactions in urban areas is a privilege granted to certain markets. If wholesalers and retailers need to trade agri-food products, they must trade in these markets. Any transaction outside the market location will be ceased by the security office. After the renovation, market management reinforced the control over wholesalers and retailers.

In addition, the renovation has expanded the domain of market management. By expanding the power of the market management, the ITB successfully established a rapid test room and entry controls in markets. In this study, we did not have the chance to interview policymakers in the local or central ITB. Nevertheless, the local ITB used to own and run the wholesale and wet markets until the 2000s. With privatization, most markets are transferred to people close to the local, public authority. From a practical perspective, for the local ITB, reaching a few market management teams is much easier than locating thousands of wholesalers and retailers. Controlling products, i.e., maintaining traceability, or controlling the producer is almost impossible for the urban ITB. Ensuring that all transactions happen in renovated markets and controlling the entry of the market seems to be a practical alternative. In addition, consumers were not consulted, and thereby they were excluded from the renovation. Because consumers were not treated as a relevant stakeholder, transparency, i.e., setting screens for the test results, was not guaranteed.

Our results show that larger wholesale markets are more successful in improving the food safety level in urban markets. We therefore recommend further concentration in the wholesale sector and increasing the scale of wholesale markets to allow a more targeted and controlled roll-out of the market renovation program. Furthermore, we have shown that successful wholesale markets benefit from close collaboration between the market management and the market regulatory administration. Hence, co-regulation is therefore recommended as a guideline for future reforms of food safety regulations, especially in Southeast Asia and South Asia, where public concerns over food safety have been growing rapidly.

This study is limited by a focus on a single province in China (Shandong) and a single class of agricultural product, vegetables. We suggest implementing similar research in other regions and for other food items. Another limit of this study is that we did not interview policymakers. Therefore, we did not have access to in-depth information about the decision-making process and the reasons for the selection of markets in the renovation program. Regarding the public authority, we have interviewed four members of the safety control staff in the markets. In this way, we obtained insights in the behavioral changes at the individual level, but not at the higher administrative level. Finally, this study revealed several limits of the renovation policy. Because the vegetable production sector is highly fragmented, the traceability of products remains elusive. The renovation program might be able to leverage the preferences of wholesalers for food quality and safety. Nevertheless, this policy is unlikely to modify farmers’ behaviour. This study also reveals that the general safety level of vegetables and the main safety hazards remain problematic because still over 13% of the vegetables in wholesale and wet markets are contaminated. Although the renovation has significantly enhanced the safety level in some of the markets, the overall share of unsafe vegetables in the renovated markets has merely been reduced to 7%. This means that vegetable safety is still worrisome in urban China.

## Figures and Tables

**Table 1:** The interviews conducted in different cities and markets.

		Visited Market	Interviewed Wholesaler/Stallholder	Interviewed Market Management
Jinan		8	32	4
Renovated markets		6	24	4
	Wholesale market	4	16	3
	Wet market	2	8	1
Unrenovated markets		2	8	0
	Wholesale market	1	4	0
	Wet market	1	4	0
Qingdao		6	24	1
Renovated markets		3	12	1
	Wholesale market	2	8	1
	Wet market	1	4	0
Unrenovated markets		3	12	0
	Wholesale market	2	8	0
	Wet market	1	4	0

**Table 2:** Number (share) of unsafe vegetable samples in markets selected for renovation and other markets.

		Selected Markets ***	Other Markets
Before renovation	2016	86/600 (14.33%)	82/603 (13.60%)
	2017	45/336 (13.39%)	54/426 (12.68%)
After renovation	2018	19/255 (7.45%)	94/564 (16.67%)

Source: based on CFDA reports. Note: significance levels for Chi-Square test: *** *p* < 0.001.

**Table 3:** Number (share) of unsafe vegetable samples in markets selected for renovation and other markets in the studied cities.

	Jinan		Zibo		Weihai		Qingdao	
	Selected	Other	Selected	Other	Selected	Other	Selected	Other
Before	87/610 (14.26%)	37/177 (20.90%)	10/113 (8.85%)	9/223 (4.04%)	14/124 (11.29%)	21/131 (16.03%)	20/89 (22.47%)	69/498 (13.86%)
After	5/146 (3.42%)	27/126 (21.43%)	2/49 (4.08%)	13/170 (7.65%)	3/14 (21.43%)	8/27 (29.63%)	9/46 (19.57%)	46/241 (19.09%)

Source: based on CFDA reports.

**Table 4:** Participation in the co-regulation processes.

	Standards Setting	Process Implementation	Enforcement	Monitoring
ITB	yes	yes	no	yes
Market Management	unknown	yes	yes	yes
Wholesaler	no	no	no	no

**Table 5:** Number (share) of unsafe vegetable samples in wholesale and wet markets selected for renovation and other markets.

	Wholesale Market ***		Wet Market	
	Selected Market	Other Market	Selected Market	Other Market
Before	87/637 (13.66%)	93/772 (12.05%)	44/299 (14.72%)	43/257 (16.73%)
After	4/117 (3.42%)	31/275 (11.27%)	15/138 (10.87%)	63/289 (21.80%)

Note: significance levels for Chi-Square test: *** *p* < 0.001.

## Appendix A

**Table A1:** Detection rate and relative size of the samples.

	Total	Detected	Detection Rate	Treatment Group	Control Group
City					
Jinan	1059	156	14.73%	757	302
Zibo	556	34	6.12%	163	393
Weihai	296	46	15.50%	138	158
Qingdao	874	144	16.48%	135	739
Market type					
Wholesale market	1801	215	11.93%	754	1047
Wet market	1417	237	16.73%	700	716
Year					
2016	1203	168	13.97%	600	603
2017	762	99	12.99%	336	426
2018	819	113	13.80%	255	564

Source: based on CFDA reports.
